# Anxiety, Depression, Coronary Artery Disease and Diabetes Mellitus; An Association Study in Ghaem Hospital, Iran

**DOI:** 10.5812/ircmj.14589

**Published:** 2014-08-17

**Authors:** Mohammad Tajfard, Majid Ghayour Mobarhan, Hamid Reza Rahimi, Mohsen Mouhebati, Habibollah Esmaeily, Gordon A Ferns, Latiffah A Latiff, Ali Taghipour, Naghmeh Mokhber, Ahmad Fazli Abdul-Aziz

**Affiliations:** 1 Department of Community Health, Faculty of Medicine and Health Sciences, University Putra Malaysia, Kuala Lumpur, Malaysia; 2 Health Sciences Research Center, Department of Health and Management, School of Health, Mashhad University of Medical Sciences, Mashhad, IR Iran; 3 Cardiovascular Research Center, Avicenna (Bu-Ali) Research Institute, Faculty of Medicine, Mashhad University of Medical Sciences, Mashhad, IR Iran; 4 Student Research Committee, Department of Modern Sciences and Technologies, Faculty of Medicine, Mashhad University of Medical Sciences, Mashhad, IR Iran; 5 Department of Cardiology, School of Medicine, Mashhad University of Medical Sciences, Mashhad, IR Iran; 6 Health Sciences Research Center, Department of Biostatistics, School of Health, Mashhad University of Medical Sciences, Mashhad, IR Iran; 7 Brighton and Sussex Medical School,University of Sussex, Brighton, UK; 8 Psychiatry and Behavioral Sciences Research Center, Faculty of Medicine, Mashhad University of Medical Sciences, Mashhad, IR Iran; 9 Department of Medicine, Faculty of Medicine and Health Sciences, University Putra Malaysia, Selangor, Malaysia

**Keywords:** Coronary Artery Disease, Anxiety, Depression, Angiography

## Abstract

**Background:**

There is an increasing trend in the prevalence of coronary artery disease (CAD) in Iran.

**Objectives:**

The present study aimed to investigate the relationship of anxiety, depression, diabetes and coronary artery disease among patients undergoing angiography in Ghaem Hospital, Mashhad, Iran.

**Patients and Methods:**

This case-control study was conducted between September 2011 and August 2012 among 200 patients undergoing coronary angiography for symptoms of coronary disease at Ghaem Hospital, Mashhad, Iran. The control group consisted of 697 healthy adults recruited from the individuals who attended the clinic for routine medical checkups or pre-employment examinations. The Beck anxiety and depression inventory scores and fasting blood glucose results were assessed in all the subjects. Data were analyzed using SPSS version 16. P < 0.05 was regarded as statistically significant.

**Results:**

The mean age of patients was 57.52 ± 9.33 years old and for the control group it was 55.35 ± 8.45 years; there was no significant difference between the subjects (P = 0.647) regarding age. There was also no significant difference in gender distribution between the patients and control groups (P = 0.205). There was however a significant difference in anxiety and depression scores between the patients and healthy controls (P < 0.001). There was a significant positive correlation between anxiety score and depression score in both groups when data were analyzed by Pearson test. (P < 0.001, r = 0.604 and r = 0.521). Moreover, there was a significant positive linear correlation between the depression/anxiety scores and fasting blood glucose concentrations in the patients group (r = 0.3, P < 0.001) and a weak negative correlation in the healthy controls (r = -0.096, P < 0.05).

**Conclusions:**

Depression and anxiety are potentially important factors among patients with angiographically-defined CAD. There appear to be significant associations between glucose tolerance and anxiety and depression in these patients.

## 1. Background

Coronary artery disease (CAD) is a major global problem. In addition, it has a higher risk of mortality for women than men. CAD has different risk factors such as lifestyle, psychological factors, environment (1, 2), age, emotional status, and smoking (3).

Depression and anxiety are common conditions affecting the general population (3), but few studies have reported the effects of depression and anxiety on general cardiac health (4-6). Anxiety sensitivity (AS) is different between individuals and its symptoms are associated with anxiety arousal. It has a negative somatic effect particularly on those who are psychologically vulnerable such as those with depression or depressive symptoms (7). Studies show that women have a higher prevalence of these symptoms than men (3).

Hadi N et al. showed, depression and anxiety were not different between breast cancer patients and control group, but they found a significant difference for anger score in their study (8).

Palizgir M and her colleagues found higher prevalence depression and anxiety levels in diabetic patients (70.7% had depression and 69.6% had anxiety) (9). Severe depression and anxiety are accompanied by immune dysregulation (10-13). Systemic cytokine concentrations can be affected by the neuroendocrine system (14) and are also under the control of corticosteroid, secreted by the adrenal cortex (15).

Plasma concentrations of the proinflammatory cytokines IL-1 and IL-6 are increased in patients with depression (16) and antidepressant medication reduces the concentrations of cytokines such as IL-1β (17). Some studies have reported a positive correlation between levels of anxiety and inflammatory cytokines (TNF-α, IL-6, and CRP) (18, 19). Depression is thought to be an important risk factor for heart diseases (20) and mood state has been identified as a determinant of quality of life in those with coronary disease (21). One in five coronary disease patients is reported to have depressive symptoms (4, 22, 23). CAD reduces the quality of life in patients; for example, it decreases their performance of everyday life activities as well as the ability to comply with their medication and diet therapies (22). The degree of depression and anxiety are also associated with a greater decline in physical functioning of patient with heart failure (4). Depression and anxiety appear to be highly overlapping with each other and they comprise more than 80% of the increased risk of CVD, adjusted for other risk factors (24). Furthermore, coronary heart diseases may lead to depression (25). Diabetes mellitus has a key role in cardiovascular diseases initiation and progression and is associated with inflammation and immune system change (26).

## 2. Objectives

To our knowledge, there is a little data on the association between mood, CAD, and fasting blood glucose; therefore, we aimed to investigate the association between anxiety, depression, and fasting blood glucose in patients with angiographically-defined CAD.

## 3. Patients and Methods

### 3.1. Participants and Procedure

This case-control study started in September 2011 and concluded in August of 2012 in Mashhad (northeast of Iran). A total of 897 subjects (466 males and 431 females) were enrolled in this study. None of the patients had a past clinical history of angiography or heart surgery and they were all > 18 years old. The cardiac patients (n = 200) underwent coronary angiography for stable angina and had at least one objective test of myocardial ischemia, for example a Dobutamin stress or exercise stress test (27). Coronary angiograms were taken using routine procedures and were all performed by a cardiologist in Ghaem Educational Hospital in northeast of Iran. We used Multi-stage cluster sampling as a random method was conducted in cardiology clinic of Ghaem hospital for the proposed study as a case control design. Since the study cardiologist only worked three days a week in Ghaem Hospital, all the cases referred to him for angiogram were screened according to the inclusion and exclusion criteria.

A total of 697 healthy controls (370 males and 327 females) were selected from people who attended clinics for routine medical check-ups or pre-employment medical examinations. The inclusion criteria of the healthy group were: adult (≥ 18), understanding the study procedures and agreeing to participate in the study, being able and willing to provide a written informed consent, being in a good health condition based on the examination, no symptoms of heart disease, no pregnancy or breast-feeding, and no history of hospitalization for any illness during the past five years (28, 29).

All the subjects in the patient group had FBG > 126 mg/dL and the healthy subjects had FBG < 126 mg/dL. All the subjects completed full questionnaires about their mental health, smoking habit, and their depression and anxiety status. If a subject had any history of drug-affected mood or chronic disease, she/he was excluded from the study. None of the subjects had a history of steroids in their drug consumption histories.

The following formula (Daniel, 1999) was used to determine the sample size:

N = (Z_α_ + Z_1-β_)^2^ (S_1_^2^ + S_2_^2^)/d^2^

Z_α_ = 0.01 is 2.81, Z_β_ = 0.10 is 1.28, test power is 90%, and S1 and S2 are standard deviations of groups 1 and 2. Based on the above formula, a minimum size of 80 sample was determined and 30% (24) was added to cater for nonresponders. The number of sample needed to be recruited was n = 80 + 24 = 104 sample per group. However, to gain a higher validity and debate the subgroups to the society and also because of the probability of the sampling, the number of sample in each group increased to two times for case and seven times for control groups.

### 3.2. Laboratory Measurement of Diabetes Mellitus

Fasted blood samples (5 mL) were collected in plain Vacutainer™ tubes for fasting blood glucose test (the tubes contained fluoride-oxalate) (30). Glucose level were measured by routine techniques, using a Cobas auto-analyzer system (ABX Diagnostics, Montpellier, France) (30).

### 3.3. Assessment of Depression and Anxiety

Beck anxiety inventory (BAI), a 21-questions multiple-choice self-report inventory, was used for measuring the severity of an individual’s anxiety (31). It assesses two factors: somatic, including 12 items explaining physiological symptoms such as “numbness or tingling”, “feeling dizzy or lightheaded”, and subjective anxiety and panic that consisted the remaining nine items of the BAI measures, such as “fear of the worst happening” and “unable to relax” (31). Each question has a 4-point score, in which 0 means not at all, 1 weak, 2 minimal, and 3 most of the time, BAI has a maximum score of 63 and 0-7 minimal anxiety, 8-15 mild anxiety, 16-25 moderate anxiety, and 26-63 severe anxiety (32, 33). Beck depression inventory (BDI), a 21-item interview, measuring the characteristic attitudes and symptoms of depression, was invented by Beck. Beck developed a triad of negative cognitions about the world, the future and the self, which plays a major role in depression. BDI has a maximum score of 63 and 0-15 indicates healthy, 16-30 minimal level of depression, 31-46 mild depression, and 47-63 severe depression (33). These questionnaires were provided to subjects before any procedure. According to Kaviani et al. study in Iran, Cronbach’s α = 0.92 as well as an acceptable test-retest reliability (r = 0.72) were found (34). Some other studies reported similar results (35). Vasegh et al. used the same Persian Beck anxiety questionnaire to the pervious report (36). The Persian format of Beck depression questioner was used for depression assessment in this study and its validity and reliability had been checked before by some researchers (Cronbach’s α = 0.87 and an acceptable test-retest reliability [r = 0.74]) (35-38). One person fielded all the questionnaires.

### 3.4. Statistical Analysis

Statistical analysis was performed using the statistical package for social sciences (SPSS) version 16. Kolmogorov-Smirnov test was used to assess the normality. Descriptive statistics (frequency, mean, and standard deviation) were determined for all the variables. Values were reported as mean ± SD for normally distributed variables (or median and IQR for non-normal distributed variables). Baseline demographics and clinical characteristics were compared among groups using student t-test, chi-square, and/or Fisher exact tests, as appropriate. Pearson correlation test was used for quantitative variables. The missing data were analyzed using appropriate statistical methods. P < 0.05 was regarded as statistically significant

## 4. Results

In this study, a total of 897 subjects were evaluated for depression, anxiety, CAD, and fasting blood glucose level. Two hundred individuals had a history of coronary disease and had angiographically-defined coronary disease and 697 were healthy subjects. The mean age of the patients’ group was 57.52 ± 9.33 years and that of the control group was 55.35 ± 8.45 years old; there was no significant difference between the subjects (P = 0.647) regarding age. There was no significant difference in gender distribution of subjects between the groups (P = 0.205) (Table 1).

**Table 1.** Characteristics of Patients and Control Groups ^a^

Variable ^b^	Patient (n = 200)	Healthy, (n = 697)	P Value
**Age, y**			0.692 ^c^
18-39	7 (3.5)	18 (2.6)	
40-59	87 (43.5)	320 (45.9)	
> 60	106 (53.0)	359 (51.5)	
**Gender**			0.205 ^c^
Male	96 (48.0)	370 (53.0)	
Female	104 (52.0)	327 (47.0)	
**Marital status**			0.007 ^c^
Single	2 (1.0)	5 (0.7)	
Married	169 (84.5)	634 (91.0)	
Divorced	1 (0.5)	11 (1.6)	
Widow/Widower	28 (14.0)	47 (6.7)	
**Education level**			< 0.001 ^c^
Primary school	164 (82.0)	439 (63.0)	
High school	26 (13.0)	168 (24.1)	
Bachelor	8 (4.0)	82 (11.8)	
Master	2 (1.0)	5 (0.7)	
Doctorate	0 (0)	3 (0.4)	
**Occupation**			< 0.001 ^c^
Employed	12 (6.0)	168 (24.1)	
Self employed	100 (50)	203 (29.1)	
Unemployed	1 (0.5)	4 (0.6)	
Student	1 (0.5)	0 (0)	
Retired	7 (3.5)	41 (5.9)	
Housewife	79 (39.5)	281 (40.3)	
**FBG, mg/dL, median (IQR)**	148 (56)	81 (15)	< 0.001 ^d^
**Anxiety score, median (IQR)**	8 (12)	5 (8)	< 0.001 ^d^
**Anxiety status**			< 0.001 ^c^
Minimal	95 (47.5)	444 (63.7)	
Mild	57 (28.5)	171 (24.5)	
Moderate	29 (14.5)	65 (9.3)	
Severe	19 (9.5)	17 (2.4)	
**Depression score, median (IQR)**	9 (12)	8 (9)	< 0.001 ^d^
**Depression status**			< 0.001 ^c^
No depression	158 (79.0)	618 (88.7)	
Mild	39 (19.5)	79 (11.3)	
Moderate	3 (1.5)	0 (0)	

^a^ Abbreviation: FBG, fasting blood Glucose.

^b^ Data are presented as No. (%).

^c^ Chi square test was used.

^d^ Independent Mann-Whitney test was used.

There was a significant difference between the groups in anxiety score (P < 0.001) and depression score, assessed by BDI (P < 0.001) (Table 1). There was also a significant difference among patient and healthy subjects in score of anxiety and in the number of subjects with no anxiety or minimal to severe anxiety scores (P < 0.001).

**Figure 1.**
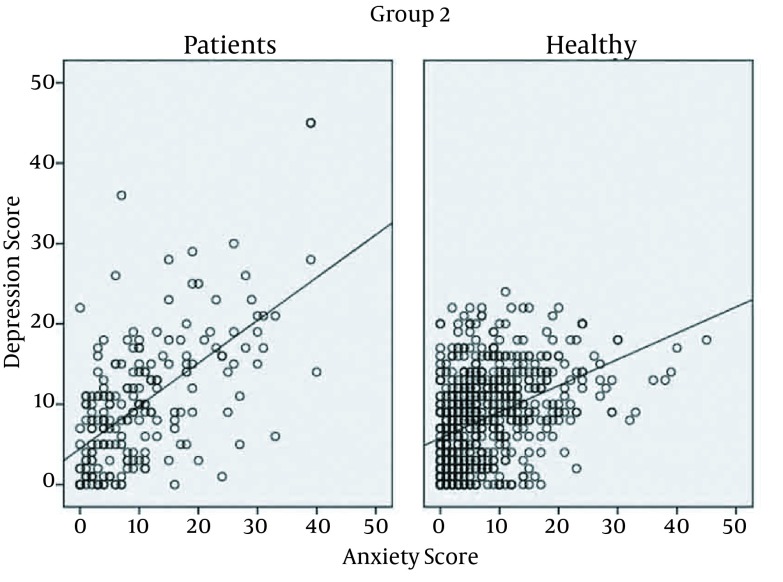
Correlation Between Anxiety and Depression Score in Healthy and Patient Groups Anxiety and depression scores are calculated by Beck tests. r = 0.302 for anxiety and 0.320 for depression. P value for both correlations is < 0.001.

**Figure 2.**
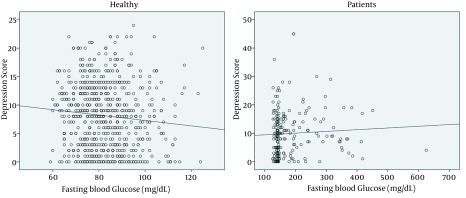
Correlation Between Depression Score and Fasting Blood Glucose in Healthy and Patient Groups Depression score is calculated by Beck test. r = -0.096 for control and 0.1 for patients' groups. P = 0.01 for control and 0.041 for patients' groups.

**Figure 3.**
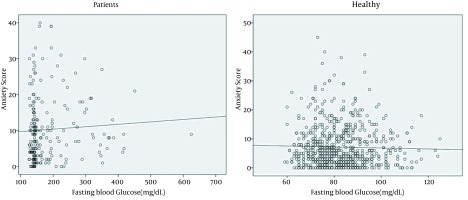
Correlation Between Anxiety Score and Fasting Blood Glucose in Healthy and Patient Groups Anxiety score is calculated by Beck test. r = -0.027 for control and 0.1 for patients' groups. P = 0.04 for control and 0.041 for patients' groups.

A positive linear correlation was found between the anxiety score and the depression score (Pearson correlation = 0.604, P < 0.001) (Figure 1). This positive correlation was found in both healthy and patient groups. There were significant correlations between fasting blood glucose and anxiety and depression score (P < 0.001 for both, Pearson correlation scores = 0.302 and 0.320 for anxiety and depression, respectively) (Figures 2 and 3).

## 5. Discussion

Depressed individuals with initially good medical health have an elevated incidence of coronary heart disease. In addition, risk of mortality is increased in people who are depressed after myocardial infarction (MI). Depression is also associated with elevated expression of inflammatory biomarkers (39).

According to a recent meta-analysis, a high level of perceived stress is associated with risk of CAD (40). Individuals with anxiety disorders or depressed moods are more prone to have unhealthy lifestyles (41). This unhealthy lifestyle is likely to be associated with exacerbation of cardiovascular risk factors such as inactivity, smoking, and unhealthy nutrition (41). In the current study, high scores for anxiety and depression were found in subjects who had angiographically-defined cardiovascular diseases, as also reported in some other studies (39, 42).

There might be a positive and unfavorable feedback between depression, anxiety, and atherosclerotic progression. This positive feedback may be mediated by an increase in circulating proinflammatory cytokines, influencing plaque progression (43, 44) and sickness behavior due to cytokine secretion. Sickness behavior can lead to inactive depressed life style which is one of the risk factors for CAD. Diabetes mellitus is a risk factor for CAD for several reasons, including enhanced NF-ĸB inflammatory signaling (43). Development of depression is also associated with elevated circulating concentrations of inflammatory biomarkers; for example, proinflammatory and antiviral cytokines (IL-2, TNF- α and IFN- α), have been associated with flu-like and depressive symptoms (14). On the other hand, TNF-α and IL-2 may be potential markers for prediction of cardiovascular events (45). Immune activation is associated with depression and increased number of circulating leucocytes and proinflammatory cytokines, such as IL-1, IL-2 and IL-6 (16, 46). In some studies, elevated serum IL-6 levels in diabetic patients and prediabetic ones has been shown (26, 47). In our study, there was a significant difference between healthy subjects and patients in depression and anxiety; a measure of glucose tolerance, fasting blood glucose concentration, was positively associated with measures of depression and anxiety.

In a recent study in Iran, it was relevant that depression and anxiety had higher prevalence among diabetic patients (9). These finding might have been due to an unfavorable positive feedback process involving psychoneuroimmunoendocrinology, CAD, and diabetes mellitus. Mental disorder may lead to inactivity and cytokine secretion. Cytokine secretion may lead to atherosclerosis plaque progression. Our control group was selected from subjects who referred for annual check-ups or pre-employment medical examinations; it may be a potential source of selection bias. Therefore, selection of the healthy group made some basic demographics differences between the groups, which was one of our limitations.

In conclusion, depression and anxiety scores are strongly related in healthy subjects and patients with CAD. There was also a significant relationship between blood glucose concentrations and these scores in patients with angiographically-defined CAD.
